# Prevalence and molecular characterization of *Cryptosporidium* spp. and *Giardia duodenalis* in dairy cattle in Gansu, northwest China

**DOI:** 10.1051/parasite/2020058

**Published:** 2020-11-18

**Authors:** Yilin Wang, Jianke Cao, Yankai Chang, Fuchang Yu, Sumei Zhang, Rongjun Wang, Longxian Zhang

**Affiliations:** 1 College of Veterinary Medicine, Henan Agricultural University Zhengzhou 450046 Henan PR China; 2 National International Joint Research Center for Animal Immunology Zhengzhou 450046 Henan PR China

**Keywords:** *Cryptosporidium* spp., *Giardia duodenalis*, Dairy cattle, Species, Assemblage, Prevalence

## Abstract

*Cryptosporidium* spp. and *Giardia duodenalis* are common gastrointestinal parasites with a broad range of hosts, including humans, livestock, and wildlife. To examine the infection status and assess the zoonotic potential of *Cryptosporidium* spp. and *G. duodenalis* in dairy cattle in Gansu, China, a total of 1414 fecal samples were collected from the rectum, with one sample collected from each individual animal. All the samples were tested using nested PCR based on the small subunit ribosomal RNA (*SSU* rRNA) gene of *Cryptosporidium* spp. and *G. duodenalis*. The overall infection rates of *Cryptosporidium* spp. and *Giardia duodenalis* were 4.2% (*n* = 59) and 1.0% (*n* = 14), respectively. Four *Cryptosporidium* species were identified: *C. andersoni* (*n* = 42), *C. parvum* (*n* = 12), *C. bovis* (*n* = 5), and *C. ryanae* (*n* = 1). In further analyses of subtypes of *C. parvum* isolates based on the 60 kDa glycoprotein (*gp60*) gene, five were successfully subtyped as IIdA19G1 (*n* = 4) and IIdA15G1 (*n* = 1). All 14 *G. duodenalis* isolates were identified as assemblage E using the triosephosphate isomerase (*tpi*) gene. The relatively low positive rates of *Cryptosporidium* spp. and *G. duodenalis* detected here and the predominance of non-human pathogenic species/assemblages of these parasites indicated their unique transmission dynamics in this area and the low level of threat posed to public health. However, continuous monitoring and further studies of these parasites should be conducted for the prevention and control of these pathogens.

## Abstract (fr)

*Cryptosporidium* spp. et *Giardia duodenalis* sont des parasites gastro-intestinaux communs avec une large gamme d’hôtes, y compris les humains, le bétail et la faune. Pour examiner le statut infectieux et évaluer le potentiel zoonotique de *Cryptosporidium* spp. et *G. duodenalis* chez les bovins laitiers à Gansu, Chine, un total de 1414 échantillons fécaux ont été prélevés dans le rectum, avec un échantillon prélevé individuellement sur chaque animal. Tous les échantillons ont été testés en utilisant une PCR nichée basée sur le gène de l’ARN ribosomal de petite sous-unité (ARNr SSU) de *Cryptosporidium* spp. et *G. duodenalis*. Les taux globaux d’infection par *Cryptosporidium* spp. et *Giardia duodenalis* étaient de 4,2 % (*n* = 59) et 1,0 % (*n* = 14), respectivement. Quatre espèces de *Cryptosporidium* ont été identifiées : *C. andersoni* (*n* = 42), *C. parvum* (*n* = 12), *C. bovis* (*n* = 5) et *C. ryanae* (*n* = 1). Dans d’autres analyses des sous-types d’isolats de *C. parvum* basés sur le gène de la glycoprotéine de 60 kDa (gp60), cinq ont été sous-typés avec succès comme IIdA19G1 (*n* = 4) et IIdA15G1 (*n* = 1). Les 14 isolats de *G. duodenalis* ont été identifiés comme étant l’assemblage E à l’aide du gène de la triosephosphate isomérase (*tpi*). Les taux positifs relativement faibles de *Cryptosporidium* spp. et de *G. duodenalis* détectés ici et la prédominance des espèces/assemblages non pathogènes pour les humains de ces parasites ont indiqué leur dynamique de transmission unique dans cette zone et le faible niveau de menace pour la santé publique. Cependant, une surveillance continue et des études plus approfondies de ces parasites devraient être menées pour la prévention et le contrôle de ces agents pathogènes.

## Introduction

*Cryptosporidium* spp. and *Giardia duodenalis* are common gastrointestinal parasites with a broad range of hosts, including humans, livestock, and wildlife [6]. These pathogens are spread by the fecal-oral route, and they can also be transmitted through contaminated food or water [10]. When infected with these parasites, most immunocompetent individuals are asymptomatic; however, immunocompromized individuals such as HIV-positive patients may suffer self-limiting diarrhea or a severe wasting and sometimes life-threatening disease [32].

At least 44 *Cryptosporidium* species and more than 70 genotypes have been described using molecular diagnostic tools based on the small subunit ribosomal RNA (*SSU* rRNA) gene [15, 38]. Previous studies indicate that more than 10 genotypes/species of *Cryptosporidium* spp. have been identified in dairy cattle, with *C. parvum*, *C. bovis*, *C. andersoni* and *C. ryanae* being the most common species [14]. Additionally, *C. scrofarum*, *C. felis*, *C. suis*, and *C. hominis* also have been detected in dairy cattle [27]. *Cryptosporidium parvum* has been classified into at least 19 subtype families: IIa to IIi and IIk to IIt, by means of sequencing analysis of the *gp60* gene [12, 17]. In China, only the IId subtype family is found in dairy cattle, including IIdA15G1 and IIdA19G1 subtypes [31]. In other countries, IIaA15G2R1 is the most common subtype in dairy cattle, and this subtype has also been commonly detected in humans. Other subtype families such as IId and IIl are uncommon and have only been reported in small numbers in cattle [34].

According to molecular characterization based on sequence analyses of *SSU* rRNA, *G. duodenalis* was classified into eight distinct assemblages (A–H), of which assemblages A and B can infect humans and various animals [10]. Assemblages C–H have strong host specificity: assemblages C and D mostly infect dogs; assemblages E, F, G, and H are specific to hoofed livestock, cats, rats, and seals, respectively [10]. However, human cases with Assemblages C, D, E and F have also been reported [5]. In dairy cattle, assemblages A, B, and E have been detected worldwide, and assemblage E is the primary assemblage in most countries [10, 35].

In China, dairy cattle have been regarded as a significant reservoir for human acquisition of cryptosporidiosis and giardiasis [11]. The distribution of *Cryptosporidium* spp. and *G. duodenalis* in pre-weaned dairy calves varies among different areas in China. The predominant species of *Cryptosporidium* spp. in pre-weaned calves in Ningxia is *C. parvum*, whereas in Henan and Shandong, it is *C. bovis*, and *C. andersoni* is the primary species in Shaanxi Province [17, 22, 24]. Only *G. duodenalis* assemblage E has been detected in Beijing [18], while *G. duodenalis* assemblage E (95.9%, 47/49) and assemblage E mixed with A (4.1%, 2/49) have been found in Hebei and Tianjin [16]; assemblages E (86.2%, 25/29) and B (13.8%, 4/29) have been found in the Ningxia Hui Autonomous Region (NXHAR) [17]. *Cryptosporidium* spp. and *G. duodenalis* infections in dairy cattle could not only be potential sources for humans acquiring cryptosporidiosis and giardiasis but also could cause great economic losses [10, 31].

Gansu Province, located on the northwestern plateau of China, is one of the key zones of both the ancient Silk Road and the New Silk Road Economic Belt. The main climatic feature of Gansu is arid conditions with scarce precipitation, making it one of the driest places in the world. According to official statistics, there are approximately 25.6 million people and 4.4 million dairy cattle in Gansu Province. To date, however, there have been few epidemiological investigations, and little is known about the prevalence and molecular characterization of parasites in dairy cattle in Gansu province. The purpose of the present study was to assess the prevalence and determine the species and genotypes of *Cryptosporidium* spp. and *G. duodenalis* in dairy cattle in Gansu province.

## Materials and methods

### Ethics statement

This study was conducted in accordance with the Chinese Laboratory Animal Administration Act of 1988. The research protocol was reviewed and approved by the Research Ethics Committee of Henan Agricultural University. The farmers gave permission to collect fecal samples, and no animals were injured during the collection.

### Sample collection

**Figure 1:**
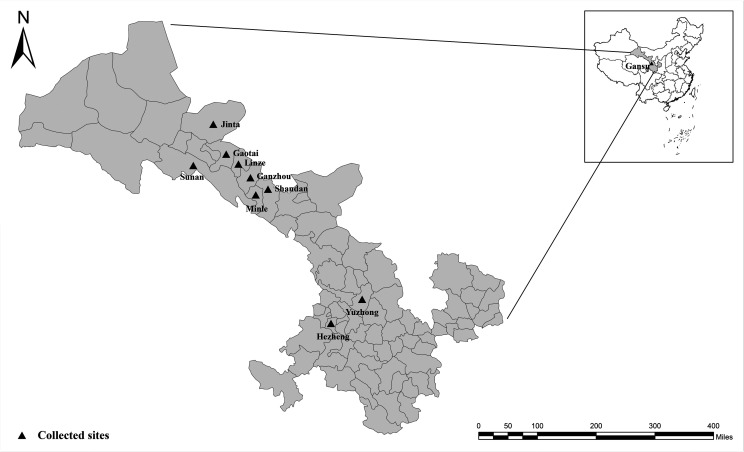
Geographic map of the sampling locations in Gansu, China. The figure was originally designed by the authors under ArcGIS 10.2 software. The original vector diagram imported in ArcGIS was adapted from Natural Earth (http://www.naturalearthdata.com).

Fresh fecal samples were collected directly from the rectum using disposable gloves, and the fecal samples were placed in containers with ice packs and quickly transported to the laboratory. All fecal samples were placed in the refrigerator at 4 °C until DNA extraction. A total of 1414 samples were collected between April 2015 and July 2015 from nine dairy cattle farms in Gansu (Fig. 1), and one sample was collected from each individual animal.

### DNA extraction

We used an E.Z.N.A.™ Stool DNA Kit (Omega Bio-Tek Inc., Norcross, GA, USA) to extract the genomic DNA, of which the first step is adding 200 mg X glass beads and 540 SLX-Mlus buffer to stool samples, then vortex at maximum speed for 10 min. The extracted DNA was stored at −20 °C before PCR amplification.

### PCR amplification

*Cryptosporidium* spp. were identified based on the small subunit (*SSU*) rRNA gene by nested PCR [37], and *C. parvum* was subtyped with nested PCR targeting the *gp60* gene [1]. *Giardia duodenalis* was identified based on the *SSU* rRNA [2] and triosephosphate isomerase (*tpi*) genes by nested PCR [4]. The primers are shown in Table 1. The products of the secondary PCR were detected by 1% agarose gel electrophoresis containing DNAGREEN (Tiandz, Inc., Beijing, China). With the Applied Biosystems 2720 Thermal Cycler (Applied Biosystems, Foster City, CA, USA), the amplifications for the *Cryptosporidium* spp. *SSU rRNA* gene and *C. parvum gp60* gene were performed in 25 μL volume, including 1 μL template DNA or primary PCR product, 2.5 μL 10× KOD-Plus PCR buffer, 2.5 μL dNTPs (2 mM), 1.5 μL MgSO_4_ (25 mM), 0.5 μL of each primer (25 μM), 16 μL double distilled water, and 0.5 μL KOD-Plus amplification enzyme (1 unit/μL) (ToYoBo Co., Ltd., Osaka, Japan). A total of 35 cycles were carried out; each of these consisted of 94 °C for 45 s, 55 °C for 45 s, and 72 °C for 1 min. There was also an initial hot start at 94 °C for 3 min and a final extension at 72 °C for 7 min. The secondary cycling conditions were identical to those used in the primary PCR. The PCR reaction mixtures for the *SSU* rRNA and *tpi* loci of *G. duodenalis* were conducted: 2.5 μL 10× PCR buffer, 2 μL dNTPs (1.25 mM), 0.3 μL each primer (25 μM), 0.2 μL rTaq DNA polymerase (1 unit/μL) (Takara Shuzo Co., Ltd), 2 μL DNA sample, 16.7 μL double distilled water, and 1 μL of bovine serum albumin (10 mg/mL). Thirty-five PCR cycles (96 °C for 45 s, 55 °C for 30 s, 72 °C for 45 s) with an initial hot start (96 °C for 4 min) and final extension (72 °C for 4 min) were carried out. The secondary PCR cycle conditions were identical to the primary round except that the annealing temperature was increased to 59 °C.

**Table 1:** Primer sequences and reaction conditions used in nested PCR amplifications.

Locus	Primer sequences (5′ – 3′)	Nucleotide fragment (bp)	Annealing temperature (°C)	Reference
*Cryptosporidium* spp. SSU rRNA	SSU-F2: TTCTAGAGCTAATACATGCG	~1325	55	Xiao et al. [37]
	SSU-R2: CCCATTTCCTTCGAAACAGGA			
	SSU-F3: GGAAGGGTTGTATTTATTAGATAAAG	826–864	55	
	SSU-R4: CTCATAAGGTGCTGAAGGAGTA			
*C. parvum gp60*	AL3531: ATAGTCTCCGCTGTATTC	1280	52	Alves et al. [1]
	AL3535: GGAAGGAACGATGTATCT			
	AL3532: TCCGCTGTATTCTCAGCC	800–850	50	
	AL3534: GCAGAGGAACCAGCATC			
*G. duodenalis* SSU rRNA	Gia2029: AAGTGTGGTGCAGACGGACTC	497	55	Appelbee et al. [2]
	Gia2150c: CTGCTGCCGTCCTTGGATGT			
	RH11: CATCCGGTCGATCCTGCC	292	59	
	RH4: AGTCGAACCCTGATTCTCCGCCCAGG			
*G. duodenalis tpi*	AL3543: AAATIATGCCTGCTCGTCG	605	50	Cacciò and Ryan [4]
	AL3546: CAAACCTTITCCGCAAACC			
	AL3544: CCCTTCATCGGIGGTAACTT	530	50	
	AL3545: GTGGCCACCACICCCGTGCC			

### Sequencing and phylogenetic analysis

The positive secondary PCR products were sequenced bidirectionally by SinoGenoMax Biotechnology Co., Ltd (Beijing, China). To confirm different species or genotypes, sequences obtained in this study were aligned with reference sequences downloaded from GenBank (http://blast.ncbi.nlm.nih.gov) using Clustal X 2.10 (http://www.clustal.org/).

To infer the phylogenetic relationships of the detected samples, neighbor-joining (NJ) trees were constructed with the MEGA X program (http://www.megasoftware.net) based on evolutionary distances calculated with the Kimura 2-parameter model. The reliability of these trees was assessed with a bootstrap analysis of 1000 replicates.

### Statistical analysis

The prevalence of parasitic infections, with the 95% confidence interval (CI), was calculated. The chi-square test was used to compare differences in infection rates between different age groups and clinical symptoms. A two-tailed *p-*value < 0.05 was considered statistically significant.

### Accession numbers

The representative nucleotide sequences obtained from this study were submitted to GenBank under the following accession numbers: ;MT820515–;MT820519, ;MT821528, and ;MT821529.

## Results

### Prevalence of *Cryptosporidium* spp. and *G. duodenalis*

Of the 1414 fecal samples taken from dairy cattle of nine farms, 60 specimens were PCR-positive for *Cryptosporidium* spp., and the overall infection rate was 4.2%. The highest infection rate (13.5%, *n* = 27) was detected on a farm in Hezheng county, followed by Minle county (5.3%, *n* = 3) and Ganzhou city (3.6%, *n* = 2). Equal infection rates (3.2%) were detected in Shandan (*n* = 4) and Jinta counties (*n* = 3), and the infection rates in Gaotai, Linze, and Sunan counties, and Yuzhong district were 2.9% (*n* = 4), 2.4% (*n* = 3), 2.3% (*n* = 9), and 2.2% (*n* = 5), respectively. Fourteen out of 1414 fecal samples were positive for *G. duodenalis,* with an overall infection rate of 1.0%. The highest infection rate (2.9%, *n* = 4) of *G. duodenalis* was observed on a farm in Gaotai, followed by Linze (2.4%, *n* = 3) and Jinta (2.1%, *n* = 2). Equal infection rates (1.8%, *n* = 1) were detected in Ganzhou and Minle, and the infection rates in Shandan and Sunan were 0.8% (*n* = 1) and 0.5% (*n* = 2), respectively. There was no *G. duodenalis* detected in the Yuzhong or Hezheng (Table 2).

**Table 2:** Species, assemblage, and genotype distribution of three enteric pathogens in cattle in Gansu.

Sampling sites	Overall infection rate (%) (no. positive/no. of samples)	Infection rate (%) (*n*)		Species/subtype/assemblage		
		*Cryptosporidium* spp.	*G. duodenalis*	*Cryptosporidium* spp.	*C. parvum*	*G. duodenalis*
Shandan	4.8 (5/125)	3.2 (4)	0.8 (1)	*C. parvum* (1), *C. andersoni* (1), *C. ryanae* (1), *C. bovis* (1)	IIdA15G2R1 (1)	Assemblage E (1)
Ganzhou	5.4 (3/55)	3.6 (2)	1.8 (1)	*C. bovis* (2)		Assemblage E (1)
Jinta	5.3 (5/94)	3.2 (3)	2.1 (2)	*C. bovis* (1), *C. parvum* (2)	IIdA19G1 (2)	Assemblage E (2)
Linze	4.8 (6/125)	2.4 (3)	2.4 (3)	*C. parvum* (1), *C. andersoni* (2)		Assemblage E (3)
Gaotai	5.8 (8/137)	2.9 (4)	2.9 (4)	*C. parvum* (4)		Assemblage E (4)
Minle	7.0 (4/57)	5.3 (3)	1.8 (1)	*C. andersoni* (2), *C. bovis* (1)		Assemblage E (1)
Yuzhong	2.2 (5/226)	2.2 (5)	0 (0)	*C. parvum* (3), *C. andersoni* (2),	IIdA19G1 (2)	
Hezheng	13.5 (27/200)	13.5 (27)	0 (0)	*C. andersoni* (26), *C. parvum* (1)		
Sunan	2.8 (11/395)	2.3 (9)	0.5 (2)	*C. andersoni* (9)		Assemblage E (2)
Total	5.2 (74/1414)	4.2 (60)	1.0 (14)	*C. andersoni* (42), *C. parvum* (12), *C. bovis* (5), *C. ryanae* (1)	IIdA15G2R1 (1), IIdA19G1 (4)	Assemblage E (14)

### Distribution of *Cryptosporidium* species/subtypes and *G. duodenalis* assemblages

By DNA sequence analysis of the *SSU* rRNA gene, the *Cryptosporidium* spp. isolates were identified as four species: *C. andersoni*, *C. parvum*, *C. bovis*, and *C. ryanae*. *Cryptosporidium andersoni* (*n* = 42), the predominant species, accounted for 71.2% of all *Cryptosporidium* spp. infections and was detected on six farms, while *C. parvum* (*n* = 12), *C. bovis* (*n* = 5), and *C. ryanae* (*n* = 1) were detected on six, three, and one farms, respectively. In the further analyses of subtypes of the 12 *C. parvum* isolates, five isolates were successfully subtyped, including subtype IIdA15G1 (*n* = 1) and subtype IIdA19G1 (*n* = 4) (Table 2).

Sequencing analyses of the *G. duodenalis SSU* rRNA gene showed that all 14 positive samples belonged to assemblage E. Two distinct genotypes from 12 assemblage E isolates were observed including E1 (*n* = 10) and E2 (*n* = 2) by amplification and sequencing of the *tpi* gene.

Concerning the ages of cattle, *C. andersoni* was found in all age groups except the <3-month group, while *C. parvum* was only found in <3-month-old dairy cattle. *Cryptosporidium bovis* and *C. ryanae* were only detected in 3–11-month-old dairy cattle. With the exception of >24-month-old dairy cattle, *G. duodenalis* assemblage E was detected in all age groups, including nine in <3-month-old dairy cattle, three in 3–11-month-old dairy cattle, and two in 12–24-month-old dairy cattle (Table 3).

**Table 3:** Infection rates and distribution of *Cryptosporidium* spp. and *Giardia duodenalis* among cattle of different ages and with or without diarrhea.

Variable	Overall infection rate (%) (no. positive/no. of samples)	Infection rate (%) (*n*) and correlation							
		*Cryptosporidium* spp.				*G. duodenalis*			
		Infection rate (%) (*n*)	*Cryptosporidium* spp.	*p*-value	OR (95% CI)	Infection rate (%) (*n*)	*G. duodenalis* assemblages	*p*-value	OR (95% CI)
Age (month)									
<3	17.5 (21/120)	10.0 (12)	*C. parvum* (12)	0.011	1.00	7.5 (9)	E (9)	<0.001	1.00
3–11	6.8 (11/162)	4.9 (8)	*C. andersoni* (3), *C. bovis* (4), *C. ryanae* (1)	0.158	0.47 (0.18–1.18)	1.8 (3)	E (3)	0.033	0.23 (0.06–0.88)
12–24	4.4 (24/546)	4.0 (22)	*C. andersoni* (22)	0.019	0.38 (0.18–0.79)	0.4 (2)	E (2)	<0.001	0.05 (0.01–0.22)
>24	2.9 (17/586)	2.9 (17)	*C. andersoni* (17)	0.001	0.27 (0.12–0.58)	0			
Symptom^a^									
With diarrhea	27.6 (16/58)	17.2 (10)	*C. parvum* (10)		1.00	10.3 (6)	E (6)		1.00
Without diarrhea	8.1 (5/62)	3.2 (2)	*C. parvum* (2)	0.014	0.16 (0.03–0.76)	4.8 (3)	E (3)	0.312	0.44 (0.10–1.85)

a Only samples from <3 month-old group are included here.

### Phylogenetic analysis

**Figure 2:**
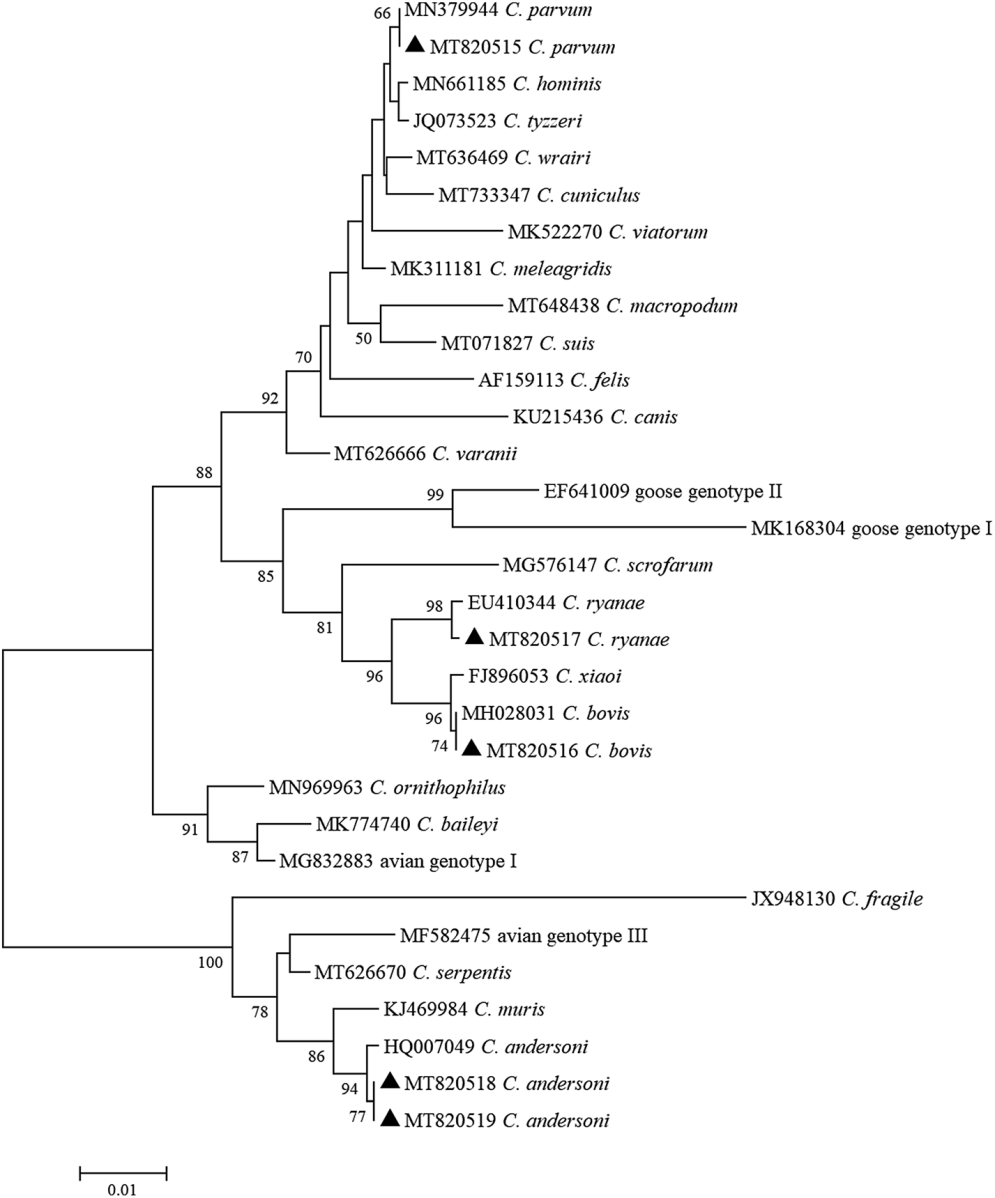
Phylogenetic tree depicting evolutionary relationships among *Cryptosporidium* spp. sequences at the SSU rRNA locus. The phylogenetic tree was inferred by a neighbor-joining analysis of genetic distances calculated by the Kimura 2-parameter mode. Percent bootstrap values greater than 50% from 1000 replicates are shown to the left of nodes. Species identified in this study are indicated by filled triangles.

**Figure 3:**
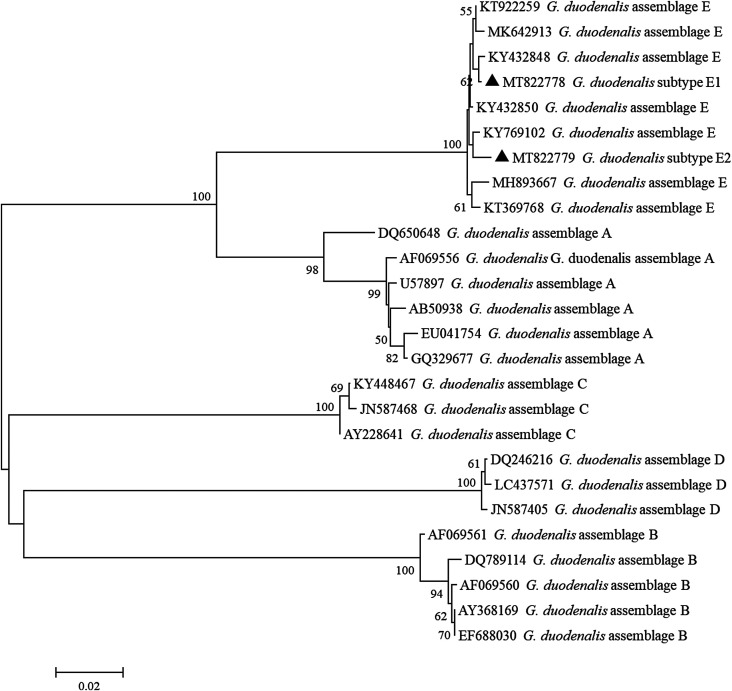
Phylogenetic tree depicting evolutionary relationships among *Giardia duodenalis* sequences at the *tpi* locus. The phylogenetic tree was inferred by a neighbor-joining analysis of genetic distances calculated by the Kimura 2-parameter mode. Percent bootstrap values greater than 50% from 1000 replicates are shown to the left of nodes. Species identified in this study are indicated by filled triangles.

From the *Cryptosporidium* spp. *SSU* rRNA locus, two nucleotide sequences of *C. andersoni* were generated and exhibited 0 and one single nucleotide polymorphism (SNP) compared to the reference sequence ;MK796098. The sequences belonging to *C. parvum*, *C. bovis*, and *C. ryanae* had 100% homology to the reference sequences ;MT002720, ;MH028031 and ;MK982468, respectively. Of the *G. duodenalis tpi* gene, E1 and E2 exhibited 100% homology to the reference sequences ;KY769101 and ;KY432851, respectively. To estimate phylogenetic inferences among the identified positive samples, NJ trees were inferred in MEGA X (Figs. 2 and 3).

### Correlation analysis

The highest *Cryptosporidium* spp. infection rate (10.0%, *n* = 12) was observed in the <3-month-old group, followed by the 3–11-month-old group (4.9%, *n* = 8), the 12–24-month-old group (4.0%, *n* = 22), and the >24-month-old group (2.9%, *n* = 17). Compared with the <3-month-old group, an OR of 0.47 (95% CI: 0.18–1.18; *p* = 0.158), an OR of 0.38 (95% CI: 0.18–0.79; *p* = 0.019), and an OR of 0.27 (95% CI: 0.12–0.58; *p* = 0.001) were associated with the 3–11-month-old group (4.9%, *n* = 8), 12–24-month-old group, and >24-month-old group, respectively. The infection rates of *G. duodenalis* were 7.5% (*n* = 9), 1.8% (*n* = 3), and 0.4% (*n* = 2) in <3-month-old, 3–11-month-old, and 12–24-month-old animals, respectively. There was a significant negative correlation between the *G. duodenalis* infection rate and age in dairy cattle, as an OR of 0.23 (95% CI: 0.06–0.88; *p* = 0.033) was associated with the 3–11-month-old group, and 0.05 (95% CI: 0.01–0.22, *p* < 0.001) was associated with the 12–24-month-old group (Table 3).

In <3-month-old dairy cattle group, a lower prevalence of *Cryptosporidium* spp. was observed in calves without diarrhea (3.2%, 2/62) compared to calves with diarrhea (17.2%, 10/58), with an OR of 0.16 (95% CI: 0.03–0.76, *p* = 0.014) (Table 3). However, the infection rates of *G. duodenalis* in <3-month-old dairy cattle group with diarrhea (10.3%, 6/58) and without diarrhea (4.8%, 3/62) were not statistically significant (*p* = 0.312) (Table 3).

## Discussion

In the present study, the overall infection rate of *Cryptosporidium* spp. was 4.2%. Similar or slightly lower infection rates have been reported in previous studies of *Cryptosporidium* spp. in dairy cattle in other provinces such as NXHAR (1.7%, 23/1366) [17], Qinghai (2.5%, 26/1027) [25], Shaanxi (2.6%, 32/1224) [42], and Gansu province (4.6%, 58/1257) [41]. The infection rate was lower than those in Shanghai (12.5%, 55/440) [7], Henan (13.0%, 276/2116) [28, 29], Anhui (14.9%, 52/350) [7], Heilongjiang (15.0%, 99/658) [20, 40], and Jiangsu (20.7%, 251/1215) [7]. In our research, the infection rate of *G. duodenalis* was 1.0%, which was consistent with the infection rates reported in NXHAR (2.1%, 29/1366) [17], and was lower than those reported in dairy cattle in Hebei and Tianjin (4.7%, 49/1040) [16], Heilongjiang (5.2%, 42/814) [21], and Jiangsu (20.6%, 281/1366) [33]. These data further confirmed that the infection rates of *Cryptosporidium* spp. and *G. duodenalis* in dairy cattle may vary according to geographical location. Moreover, water-borne transmission is an important route of *Cryptosporidium* spp. and *G. duodenalis* cross-species transmission [36], and Gansu has a unique geographical location and climatic conditions that may be the reasons for the lower infection rate in this study. Additionally, sampling time, number of samples, breeding methods, animal health status, and experimental methods also have a certain impact on the infection rate.

The dominant *Cryptosporidium* species in <3-month-old, 3–11-month-old, 12–24-month-old and > 24-month-old groups were *C. parvum*, *C. bovis*, *C. andersoni* and *C. andersoni*, respectively. Remarkably, the distribution of the four most common *Cryptosporidium* species is related to the age of dairy cattle. *Cryptosporidium parvum* is often detected in pre-weaned calves, which currently result in pre-weaned calf diarrhea. *Cryptosporidium bovis* and *C. ryanae* are commonly seen in post-weaned calves and yearlings, and *C. bovis* is more common than *C. ryanae* [26]. However, *C. andersoni* is more likely to infect adult dairy cattle, which normally leads to lower milk production and weight loss [8].

Of the 12 *C. parvum* isolates, 5 were identified as subtype IIdA19G1 (*n* = 4) or subtype IIdA15G1 (*n* = 1) based on sequencing analysis of the *gp60* gene, and these 2 subtypes have also frequently been found in bovines [31]. In addition, subtypes IIdA19G1 and IIdA15G1 have also been detected in ruminants (sheep, goats, deer), alpacas, horses, rodents (mice, hamsters, squirrels), pigs, carnivores (dogs, gray wolves, raccoon dogs), and in wastewater around the world [3, 19]. Meanwhile, previous reports have shown that subtypes IIdA15G1 and IIdA19G1 can also cause human infections [13, 30]. Thus, despite the low positive rates of these two subtypes, the threat posed to public health cannot be neglected.

In this study, *G. duodenalis* assemblage E was the dominant assemblage, consistent with the results reported worldwide [23]. These results were in accordance with the previous definition of assemblage E as a bovine-specific assemblage. However, assemblage E has also been found in several human cases in Brazil and Australia [9, 39]. Therefore, more advanced epidemiological studies are required to assess the risk of assemblage E infecting humans. It is necessary to prevent and control *G. duodenalis* transmission for the protection of animals and breeders in the area, although the zoonotic risk of *G. duodenalis* was limited in this study.

In conclusion, the infection rates of *Cryptosporidium* spp. and *G. duodenalis* were relatively low in this area. Moreover, the study revealed the presence of *C. parvum* subtypes IIdA19G1 and IIdA15G1, and only *G. duodenalis* assemblage E was identified in dairy cattle in Gansu province, indicating that zoonotic transmission of *Cryptosporidium* spp. and *G. duodenalis* is possible but unlikely. Therefore, it is necessary to adopt effective strategies to prevent and control the transmission of *Cryptosporidium* spp. and *G. duodenalis* among dairy cattle and humans in Gansu province.

## Conflict of interest

The authors declare that they have no conflicts of interest.
